# Integrins in multiple myeloma

**DOI:** 10.1186/s41232-020-00113-y

**Published:** 2020-03-30

**Authors:** Naoki Hosen

**Affiliations:** Department of Cancer Stem Cell Biology, Osaka University Graduate School of Medicine, Suita, 1-7 Yamada-Oka, Suita, Osaka, 565-0871 Japan

**Keywords:** CAR T cell, Multiple myeloma, Integrin, Protein conformation

## Abstract

Integrins have crucial roles in BM homing, survival, proliferation, or drug resistance of multiple myeloma (MM) cells. Especially, integrin α4β1 (VLA-4) and α4β7 has been reported to have important functions in MM cells, and therefore are potential therapeutic targets. We have recently shown that integrin β7 constitutively adopts the active conformation specifically in MM cells, and found that chimeric antigen receptor (CAR) T cells targeting the activated conformation of integrin β7 is promising for MM. Although the mechanism for the constitutive activation is still being investigated, our results indicate that integrin conformation is different between MM and normal cells and suggest that it may be associated with the pathology of MM.

## Integrins in multiple myeloma

Multiple myeloma (MM) cells preferentially reside in bone marrow (BM) microenvironment. Extra-cellular matrix (ECM) proteins such as fibronectin, collagen, laminin, and osteopontin form BM microenvironment together with several kinds of cells including hematopoietic cells, erythrocytes, stromal cells, endothelial cells, as well as osteoclasts and osteoblasts. Numbers of reports have shown that the interaction of MM cells with ECM proteins and accessory cells in the BM microenvironment has essential role for the survival and proliferation of MM cells.

MM cells mainly reside in BM. However, continuous egress of MM cells in the peripheral blood and reentrance into the bone marrow are evident during the progression of MM, suggesting that the potential of BM homing is important for MM cells. The homing of MM cells to BM is mediated by the chemokine CXCL12, which is produced by a subset of BM stromal cells. CXCL12 interacts with its receptor CXCR4 expressed on MM cells and induce BM homing of MM cells [1]. In BM microenvironment, MM cells adhere to either ECM proteins or BM stromal cells. Such adhesion is mediated by adhesion molecules including CD44, very late antigen 4 (VLA4), VLA5, leukocyte function-associated antigen 1 (LFA1), neural cell adhesion molecule (NCAM), intercellular adhesion molecule 1 (ICAM1), syndecan 1, and MPC1. Among these adhesion molecules, we focused on integrins in this review. Integrins are an extensive family of glycoproteins expressed by many cell types, including MM cells in which a wide range of integrins (α4, α5, αv, β1, β2, β3, and β7) is detected [2, 3]. In particular, α4 β1 (VLA-4) [4–7] and α4β7 [8] are shown to regulate MM-cell adhesion, migration, homing, invasion as well as drug resistance.

## VLA-4

VLA-4 is a heterodimer of integrin α4 and β1. VLA-4 is expressed on MM cells and mediates binding to the ECM such as fibronectin. The binding of MM cells to fibronectin upregulates p27 and induces nuclear factor κB (NFκB) activation in MM cells [6], which confer cell adhesion-mediated drug resistance (CAM-DR) to conventional chemotherapy [9]. VLA4 also mediates binding of MM cells to BMSCs through vascular cell adhesion molecule (VCAM-1). MM cell adhesion to stromal cells via VLA-4 and VCAM-1 interaction causes enhanced secretion of MIP-1α and MIP-1β, which have osteoclastogenic activity, by MM cells. It was also reported that CAM-DR induced by the adhesion of MM cells to stromal cells were dominantly mediated by VLA-4 [10].

In consistent with these important function of VLA-4 in the adhesion of MM cells, natalizumab, which is an anti-integrin α4 monoclonal antibody, inhibited adhesion of MM cells to ECM proteins or BM stromal cells. Furthermore, natalizumab can disrupt the binding of MM cells that have already adhered to ECM or stromal cells [11]. Consequently, natalizumab inhibited the proliferation of MM cells co-cultured with BM stromal cells. In addition, natilizumab blocked angiogenesis induced by vascular endothelial growth factor (VEGF) secreted as a result of MM-stromal cell interaction. Moreover, natalizumab also inhibited MM cell migration mediated by VEGF- and insulin-like growth factor 1 (IGF-1). In consistent with these in vitro results, natalizumab treatment reduced growth of MM cells in vivo. Furthermore, natalizumab treatment sensitized MM cells to bortezomib in in vitro co-culture of MM cells with BM stromal cells.

## Integrin β7

Integrin β7 forms heterodimer with either α4 or αE. Integrin β7 mRNA is expressed in MM cells. It was reported that *integrin β7* gene expression is regulated by *MAF* gene, and therefore integrin β7 is highly expressed in MM patients with elevated MAF expression, especially in those with IgH-MAF translocation. It was also reported that integrin β7 expression is regulated by KDM3A–KLF2–IRF4 axis [12]. KDM3A induces demethylation of the promoter regions of KLF2 and IRF4 and maintain their expression. Integrin β7 expression was downregulated in MM cells when KDM3A, KLF2, or IRF4 were silenced. Silencing of integrin β7 in MM cells reduces adhesion to fibronectin or E-cadherin, and inhibit CAM-DR of MM cell to bortezomib or melphalan. In addition, MM-cell transwell migration in response to CXCL12 gradients were reduced by silencing integrin β7. Consistently, integrin β7 silencing reduced homing of MM cells into BM in vivo. Integrin β7 knockdown inhibited VEGF production in MM co-cultured with BM stromal cells. In consistent with this, silencing of integrin β7 in MM cells reduced vessel density in xenograft tumors. These findings suggested that integrin β7 has crucial roles in MM-cell adhesion, migration, and BM homing.

## Integrin β7 constitutively adopts the activated conformation in MM cells

**Fig. 1:**
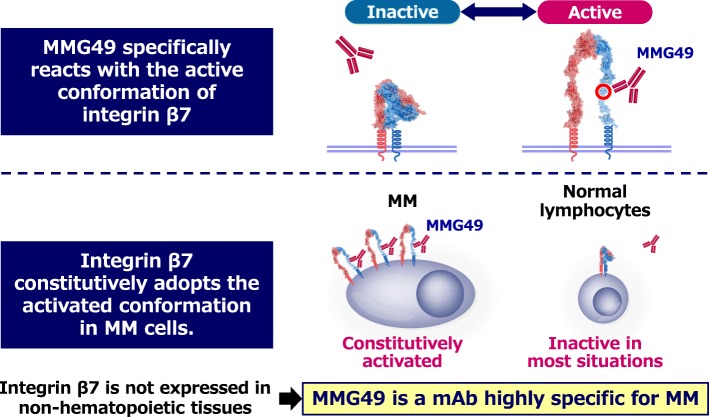
MMG49 specifically reacts with the activated conformation of integrin β7 expressed on MM cells

Integrins adopt the inactive “bent” conformation in steady state, but the active “extended” conformation upon ligand-ligation or stimulation by external factors such as chemokines. We unexpectedly found that integrin β7 constitutively adopts the active conformation in MM cells during the course of screening for MM-specific cell surface antigens. We tried to identify cell surface antigens that are specifically expressed in MM cells. Since many researches for isolating MM-specific genes or proteins have been performed using comprehensive transcriptome or proteome analyses, most MM-specific transcripts or proteins are likely to be already identified. However, cancer-specific antigens are formed by post-translational events such as glycosylation or conformational changes may have been missed. To identify such antigens, we first made numbers of monoclonal antibodies (mAbs) that react with MM cells, and then isolated MM-specific mAb among them. We established more than 10,000 clones of mAbs that react with MM cells. Next, 504 mAbs were selected as mAbs that react with MM cell lines but not with normal peripheral blood mononuclear cells. Then, we stained bone marrow (BM) cells from MM patients with those candidate mAbs and analyzed on flow cytometry. Finally, MMG49 was found to be a mAb that bound specifically to MM cells but not to CD45^+^ normal leukocytes in the BM. Next, we found that MMG49 bounds to integrin β7 protein by the expression cloning method. This result surprised us, because integrin β7 is known to be expressed on normal lymphocytes and in fact normal lymphocytes were positively stained with a pre-existing anti-integrin β7 mAb. Interestingly, MMG49 did not react with normal lymphocytes although they no doubt express integrin β7 protein. Then, we found that MMG49 binds only to the active conformation of integrin β7, but not to the inactive conformation. The MMG49 epitope is located in the N-terminal region of the β7 chain, which is predicted to be exposed in the active conformation, but not in the inactive conformation. We also demonstrated that integrin β7 constitutively adopts the active conformation. Therefore, MMG49 abundantly binds to MM cells (Fig. 1). Integrin β7 is also expressed in normal lymphocytes at low levels, but adopts the inactive conformation. Thus, MMG49 rarely binds to normal lymphocytes. In addition, integrin β7 is not expressed in non-hematopoietic tissues. These results suggest that the MMG49 antigen is a good therapeutic target for MM. We established CAR T cells using the sequences of the antigen recognition domain of MMG49. MMG49 CAR T cells eradicated MM cells in in vivo xenograft models without impairing normal hematopoiesis, suggesting that MMG49 CAR T cells targeting the activated conformation of integrin β7 is promising for MM treatment. Clinical trials are being prepared now. Importantly, these results provide the first clear evidence that a receptor protein with a rare but physiologically relevant conformation can serve as a target for CAR T cell therapy [13].

## What is the mechanism of constitutive activation of integrin β7 in MM cells?

An obvious question is why integrin β7 constitutively adopts the active conformation in MM cells, although we have not clarified it yet. Many researchers have extensively analyzed the mechanisms for the regulation of integrin activation. It is known that integrin activation is regulated by two kinds of signals. One is inside-out signaling that control the activation of integrins in response to various stimuli, for example from antigen receptors or chemokine receptors. Another is outside-in signaling induced by integrin dependent adhesion to ECM proteins or other cells. When leukocytes attached to ECM proteins or other cells, inside-out signaling induce conformational change of integrins and increase their affinity to ligands. Then, the interaction is strengthened by outside-in signaling from integrins. Several studies using gene knock-out or knock-down revealed that several molecules are involved in the inside-out signaling. The inside-out pathways that induce integrin activation are different depending on the types of cells (e.g., T and B lymphocytes, neutrophils, platelets). Nonetheless, among many molecules associated with the inside-out signaling, Rap small GTPases and talin/kindlin-3 are likely to be key players.

Several reports suggested that the small GTPase Rap1 is an important regulator of cell adhesion [14, 15]. Both Rap1a and Rap1b are expressed in most tissues at various levels. Deletion of the *rap1a or rap1b* genes in lymphocytes caused impaired activation of VLA-4 and LFA-1 [16, 17]. In addition, *rap1b* deletion resulted in impaired activation of integrin αIIbβ3 on platelets [18]. These results indicate that Rap1 has an essential role in integrin activation. Like other small GTPases, Rap1 exists as an inactive GDP-bound form or an active GTP-bound form. We examined whether active GTP-bound Rap1 increased in MM cells and caused constitutive activation of integrin β7. However, activation of Rap1 was not likely to be a major mechanism for it (unpublished data).

Talin and kindlin-3 are critical for inside-out and outside-in signaling [19]. Talin is ubiquitously expressed and links integrins to the actin cytoskeleton. Talin protein has the integrin-binding domain and the domain that binds to vinculin, which is the F-actin binding protein. When talin binds to the cytoplasmic region of β integrins, conformational changes occur and the affinity of integrins to the ligands are increased, resulting in the development of focal adhesion through integrin clustering [20]. Kindlin-3 also binds to cytoplasmic regions of β integrin, although the kindlin-3 binding domains are distinct from those involved in talin binding. Deletion of kindlin-3 in mice caused severe bleeding due to defective platelet aggregation. Kindlin-3 is also necessary for firm attachment and spreading via β2 integrins. The mechanism of talin and/or kindling-3 activation, especially in leukocytes, when stimulated with chemokines and the antigen receptors, is unclear. It has not been clarified yet whether talin or kindling-3 is associated with constitutive activation of integrin β7 in MM cells.

Integrin–ECM adhesion turnover is also regulated by endocytic trafficking of integrins. It is known that active and inactive integrin conformers can be recycled through distinct routes. Active and inactive β1 integrins are endocytosed through the same routes to early endosomes [21]. Then, active β1 is recycled through the long Rab11-dependent recycling loop, whereas the inactive β1 receptor is rapidly recycled in an actin and Rab4-dependent manner to the plasma membrane. Consequently, inactive β1 is mostly localized at the plasma membrane, while active β1 integrin tends to exist in cytoplasm. It was also reported that ligand bound active integrins traffic to late endosomes and lysosomes [22]. In late endosome or lysosome, ligands were detached from active integrins in an acidic environment, and free integrins are recycled to the plasma membrane. In consistent with these findings, recycling of active β1 integrin were reported to be slower than that of inactive ones, and the active, but not the inactive receptor, was observed in Rab7-positive late endosomes [21]. Interestingly, active integrins can be recycled to the plasma membrane directly from lysosomes without losing the active conformation in cancer cells. These results showed that recycling pathway of active integrin is different from that of inactive integrin. While it should be analyzed whether such difference is also observed in the case of integrin β7, dysregulation of active and inactive integrin β7 trafficking in MM cells may be a cause of accumulation of active integrin β7 in MM cells.

## Conclusions

Integrins, especially VLA-4 (α4β1) and α4β7, have substantial roles in the pathophysiology of MM. We have recently found that integrin β7 constitutively adopts the activated conformation, suggesting that difference in conformation between normal cells and MM cells may also be found in other integrins. We should clarify the mechanism for constitutive activation of integrin β7 in MM cells and also the functional role of the activated integrin β7 in the pathogenesis of MM.

## Data Availability

Not applicable
